# Hippocampus and amygdala radiomic biomarkers for the study of autism spectrum disorder

**DOI:** 10.1186/s12868-017-0373-0

**Published:** 2017-07-11

**Authors:** Ahmad Chaddad, Christian Desrosiers, Lama Hassan, Camel Tanougast

**Affiliations:** 1 Laboratory for Imagery, Vision and Artificial Intelligence, Ecole de Technologie Supérieure, Montreal, Canada; 2 Laboratory of Conception, Optimization and Modeling of Systems, University of Lorraine, Metz, France

**Keywords:** Autism spectrum disorder, Hippocampus, Radiomics

## Abstract

**Background:**

Emerging evidence suggests the presence of neuroanatomical abnormalities in subjects with autism spectrum disorder (ASD). Identifying anatomical correlates could thus prove useful for the automated diagnosis of ASD. Radiomic analyses based on MRI texture features have shown a great potential for characterizing differences occurring from tissue heterogeneity, and for identifying abnormalities related to these differences. However, only a limited number of studies have investigated the link between image texture and ASD. This paper proposes the study of texture features based on grey level co-occurrence matrix (GLCM) as a means for characterizing differences between ASD and development control (DC) subjects. Our study uses 64 T1-weighted MRI scans acquired from two groups of subjects: 28 typical age range subjects 4–15 years old (14 ASD and 14 DC, age-matched), and 36 non-typical age range subjects 10–24 years old (20 ASD and 16 DC). GLCM matrices are computed from manually labeled hippocampus and amygdala regions, and then encoded as texture features by applying 11 standard Haralick quantifier functions. Significance tests are performed to identify texture differences between ASD and DC subjects. An analysis using SVM and random forest classifiers is then carried out to find the most discriminative features, and use these features for classifying ASD from DC subjects.

**Results:**

Preliminary results show that all 11 features derived from the hippocampus (typical and non-typical age) and 4 features extracted from the amygdala (non-typical age) have significantly different distributions in ASD subjects compared to DC subjects, with a significance of *p* < 0.05 following Holm–Bonferroni correction. Features derived from hippocampal regions also demonstrate high discriminative power for differentiating between ASD and DC subjects, with classifier accuracy of 67.85%, sensitivity of 62.50%, specificity of 71.42%, and the area under the ROC curve (AUC) of 76.80% for age-matched subjects with typical age range.

**Conclusions:**

Results demonstrate the potential of hippocampal texture features as a biomarker for the diagnosis and characterization of ASD.

## Background

Autism spectrum disorder (ASD) is a complex developmental disability that appears during infancy, specifically the first 2–3 years of life [1, 2]. It is a spectrum disorder affecting about one in 300 children to varying degrees [3]. To this day, the exact causes of ASD are not fully understood, and it is believed that a combination of genetic and environmental factors are involved [4, 5]. Over the years, MRI has been a key technology for the in vivo study of ASD, facilitating the visualization of neuroanatomical structures related to this disorder, such as the hippocampus and amygdala. Image features, extracted from MRI data, have shown a great potential for the study of various neurological disorders like Alzheimer’s [6]. However, their application to the study of ASD, and particularly for differentiating between ASD and development control (DC) subjects, remains limited.

Previous studies have shown a link between ASD and morphological characteristics measured from MRI. For instance, children with ASD exhibit an alteration of hippocampal shape consistent with inward deformation of the subiculum [7]. Likewise, a connection has been reported between ASD and neuronal size abnormalities in medial temporal lobe structures, including the hippocampus [8]. Studies have also investigated the relationship between ASD and neuroanatomical regions other than the hippocampus, namely, the cerebral cortex, cerebellum, amygdala, corpus callosum and caudate nucleus [9–11].

Nevertheless, there are striking inconsistencies in the evidence linking ASD to volumetric abnormalities in MRI [12]. Several studies suggest that autistic children between the age of 2 and 4 have a significantly larger brain compared to normally developing peers [13–15]. An increase in hippocampal volume, persisting to adolescence, has also been reported in the literature [16]. However, other studies involving autistic adolescents and young adults showed no significant difference [17], or even a decrease in hippocampal volume [18]. Likewise, the orbitofrontal cortical thickness of ASD subjects was found to be enlarged in Ecker et al. [10], while decreased in other studies [11, 19]. Inconsistencies between these ASD studies suggest that the neuroanatomical correlates of this complex disorder are quite variable. This variability may also arise due to differences in the mode (or equipment/site) of imaging data acquisition and analysis.

So far, studies have mostly focused on volumetric (or thickness) features derived from MRI images, and have not fully exploited the rich information captured by radiomic features. Radiomic analyses focus on the automated and high-throughput extraction of features from medical images, which captures subtle changes in regions of interest and quantifies patterns which might escape the human visual system [20]. In particular, the texture features studied in such analyses provide an intuitive means for characterizing general image heterogeneity in MRI. They also offer a powerful way of detecting various diseases, such as Alzheimer’s [6], glioblastoma [21] and colon cancer [22].

In a recent study, Chaddad et al. found significant texture differences between the MRI scans of ASD and DC subjects, occurring predominantly in the hippocampus [23]. These differences suggest that texture features could be used as biomarkers for ASD diagnosis, complementary to traditional morphological measurements like volume. Based on these recent findings, this work proposes using radiomic features, extracted from the hippocampus and amygdala regions in MR images, to differentiate ASD from DC subjects. To our knowledge, this is the first study to use texture features effectively for this task.

We present an automatic processing pipeline based on texture feature extraction, region encoding and subject classification. Specifically, we investigate 11 different texture features derived from grey-level co-occurrence matrices (GLCM), which have been used successfully in previous studies [24–26]. GLCM features are extracted from segmented hippocampi and amygdala regions in MRI, these two neuroanatomical structures linked to memory formation and believed to play a role in ASD. For instance, the amygdala could be connected to socio-emotional impairments in ASD [27, 28]. These features are then employed within an analysis of variance (ANOVA) test, and used as inputs to support vector machine (SVM) and random forest models for identifying dominant texture features that can reliably differentiate between ASD and DC subjects.

## Methods

**Fig. 1:**
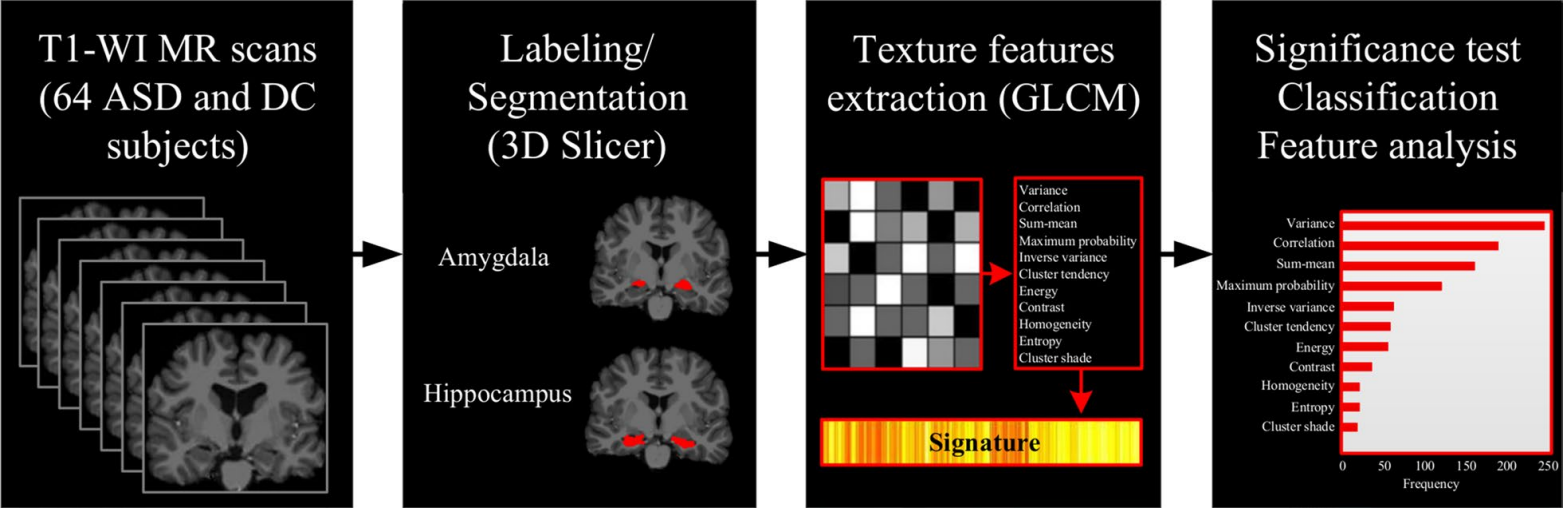
Workflow of the proposed model. Data derived from T1-weighted MRI [scans reproduced with permission from the International Neuroimaging Data-Sharing Initiative (INDI), under the creative commons license (https://creativecommons.org/licenses/by-nc-sa/3.0/)]; manual labeling of hippocampus and amygdala regions; extraction of GLCM-based texture features from hippocampus and amygdala regions; identification of discriminative features for classifying ASD and DC subjects

The flowchart of the proposed method, shown in Fig. 1, comprises three steps: (1) region labeling, (2) GLCM feature extraction and (3) ASD versus DC classification. The data used in our study and these steps are discussed in following sub-sections.

### Patient population and data acquisition

MRI images of 64 subjects were obtained from the publicly available ABIDE I database [29, 30]. This database consists of structural MRI provided by various medical and research sites around the world. Diagnosis of ASD was achieved using the Autism Diagnostic Observation Schedule (ADOS), the Autism Diagnostic Interview-Revised (ADI-R), or both [31, 32]. The imaging protocol used was whole-brain T1-WI scanning with a 3T MRI scanner. All volumetric images were acquired with a resolution of 1 mm^3^, for a total size of 256 × 256 × 256 voxels [29, 30]. In accordance with Health Insurance Portability and Accountability (HIPAA) guidelines, all data are anonymized with no protected health information included.

We considered the following two subject groups: (A) typical age range children, further divided into (A1) 14 children with ASD (6 males, 8 females; median age 12.87-year-old; range 4–15 years) and (A2) 14 children with DC (6 males, 8 females; median age 13.97 year-old; range 4–15 years), having similar range of demographics; (B) non-typical age range subjects, divided into B1) with 20 ASD subjects (17 males, 3 females; median age 17 year-old; range 11–24 years) and (B2) 16 DC subjects (14 males, 2 females; median age 16.5 year-old; range 10–23 years). The 28 subjects in group A were selected from the University of Michigan (UM) site of the ABIDE I database. This enables us to have balanced data (ASD/DC samples) and avoid inter-site variations resulting from differences in acquisition equipment or protocol. Within this group, the 6 males and 8 females with ASD were individually matched based on age with the 6 males and 8 females labeled as DC. The 36 subjects in group B were taken from the University of Pittsburgh (Pitt) site, the remaining 27 subjects of this site ignored due to poor labelling quality (>2 mm error). Subjects in this unbalanced group (20 ASD vs 16 DC) were not age-matched, allowing us to evaluate the effect of this confound in our analysis. A detailed description of our study’s final sample is provided in Table 1.

**Table 1:** Demographic and clinical characteristics of the study groups

Subjects	Study group	n	Sex (male/female)	Age (years)	*p* value* (male vs female)	*p* value* (ASD vs DC)
Group A	ASD	14	6/8	12.87 (4–15)	0.791	0.886
	DC	14	6/8	13.97 (4–15)	0.811	
Group B	ASD	20	17/3	17.00 (11–24)	0.021	0.835
	DC	16	14/2	16.50 (10–23)	0.013	

* *p* value of age subjects

### Region labeling

Hippocampi and amygdala were labeled manually, in a blind fashion, by two independent expert radiologists with 5 and 7 years of experience. The segmentation was performed slice by slice from sagittal images of T1-WI MRI scans, using the open source 3D Slicer 3.6 platform (http://www.slicer.org/). The inter-rater reliability of the segmentation was measured using the Dice similarity coefficient (DSC) [33] which evaluates the degree of correspondence between two labels (i.e., a labeling from the first expert compared to that of the second expert).

**Fig. 2:**
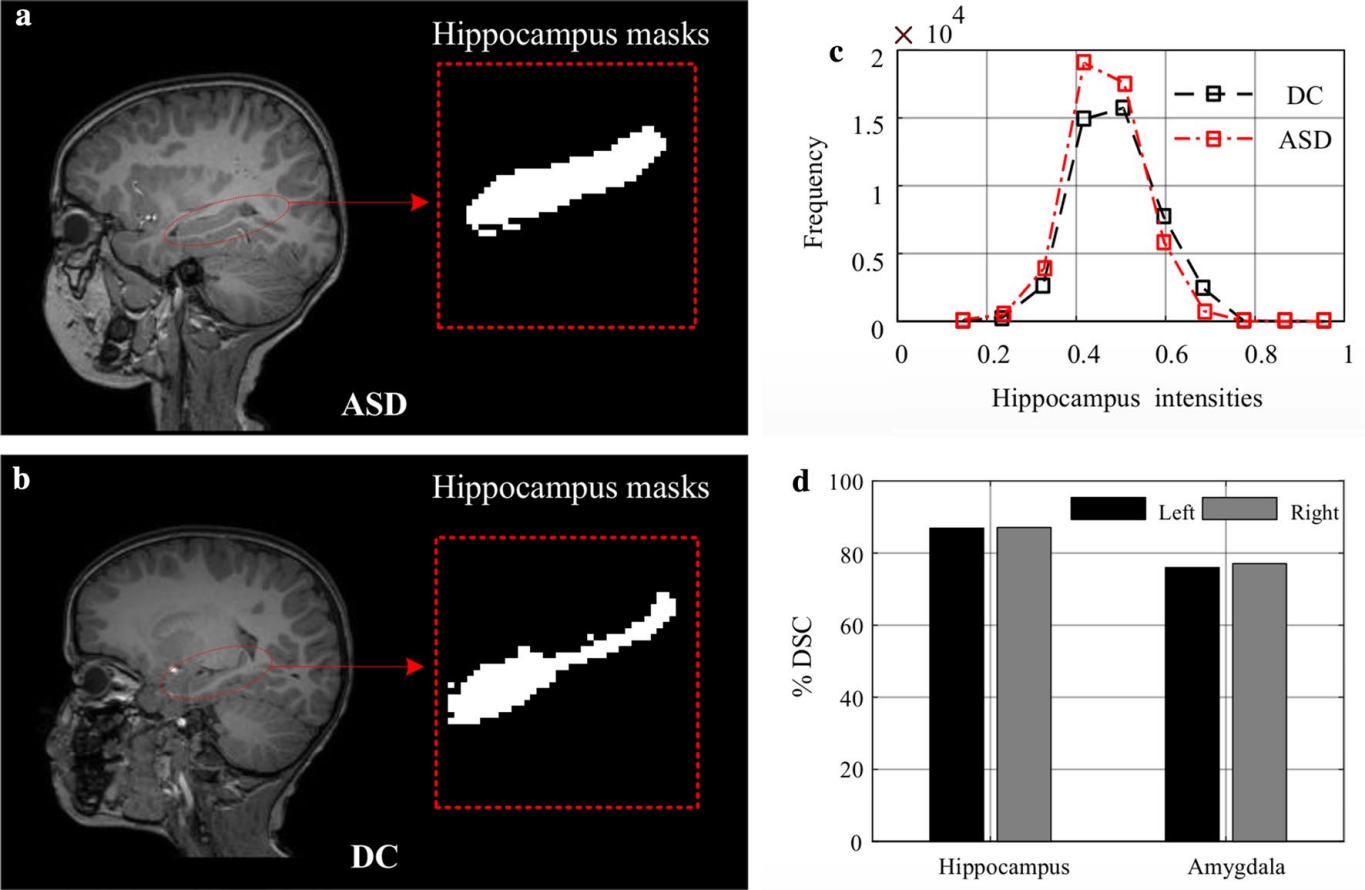
Examples of hippocampus regions in ASD and DC subjects. **a**, **b** Examples of hippocampus segmentation masks for ASD and DC subjects (scans reproduced with permission from the International Neuroimaging Data-Sharing Initiative (INDI), under the creative commons license); **c** histogram of normalized intensities in MRI images of ASD and DC typical age range subjects; **d** dice similarity coefficient between the two expert labelings (*left* and *right* of hippocampus and amygdala regions, respectively)

Figure 2 shows examples of hippocampal labels in ASD (Fig. 2a) and DC (Fig. 2b) subjects. The histogram of normalized intensities derived from ASD and DC subjects in group A are given in Fig. 2b. We see that these histograms are similar to one another and, therefore, that raw intensity values in the hippocampus are not reliable for differentiating between ASD and DC subjects. More informative features, such as those encoding texture, are thus necessary to the capture the subtle differences arising from ASD. Toward this goal, we used the segmentation masks of hippocampus and amygdala regions to extract texture features based on GLCM.

### GLCM based texture features

Neighboring pixels are known to exhibit correlation in natural images. As proposed in the seminal work of Haralick in 1973 [34], local variations in an image captured by GLCMs can be used effectively to characterize the image’s texture. GLCMs are second-order statistics which estimate the properties of two or more pixel values occurring at specific locations relative to each other. Specifically, GLCM entries correspond to the co-occurrence probability *P*_*d*,*θ*_(*i*, *j*) of having intensities *i* and *j* in two pixels separated by a translation vector defined using direction *θ* and offset *d* (also known as *distance*) [11, 16–19]. Given a 2D image *I* of size *N* × *N*, the co-occurrence matrix *P*_*d*,*θ*_(*i*, *j*) can be defined element-wise as

1 $$P_{d,\theta } \left( {i,j} \right) = \mathop \sum \limits_{x = 1}^{N} \mathop \sum \limits_{y = 1}^{N} \left\{ {\begin{array}{*{20}l} {1,} \hfill & {\quad {\text{if}}\;I(x,y) = i\; {\text{and}}\;I\left( {x + dx, y + dy} \right) = j} \hfill \\ {0,} \hfill & {\quad {\text{otherwise}} } \hfill \\ \end{array} } \right.$$

where *dx* and *dy* are the translations along the *x*-axis and *y*-axis, corresponding to direction *θ* and offset *d*. A GLCM is thus a square matrix of size *Ng*, where *Ng* is the number of grey levels in the image. For 2D images, a total of 16 GLCMs are typically computed, each one corresponding to the combination of an offset *d* ∈ {1, 2, 3, 4} and a direction *θ* ∈ {0°, 45°, 90°, 135°}. Note that directions {180°, 225°, 270°, 315°} are redundant because of the symmetry found in GLCM matrices: *P*_*d*,0°_ = *P*_*d*,180°_^T^, *P*_*d*,45°_ = *P*_*d*,225°_^T^, *P*_*d*,90°_ = *P*_*d*,270°_^T^, *P*_*d*,135°_ = *P*_*d*,315°_^T^, where the superscript ‘T’ denotes the transpose operation.

**Fig. 3:**
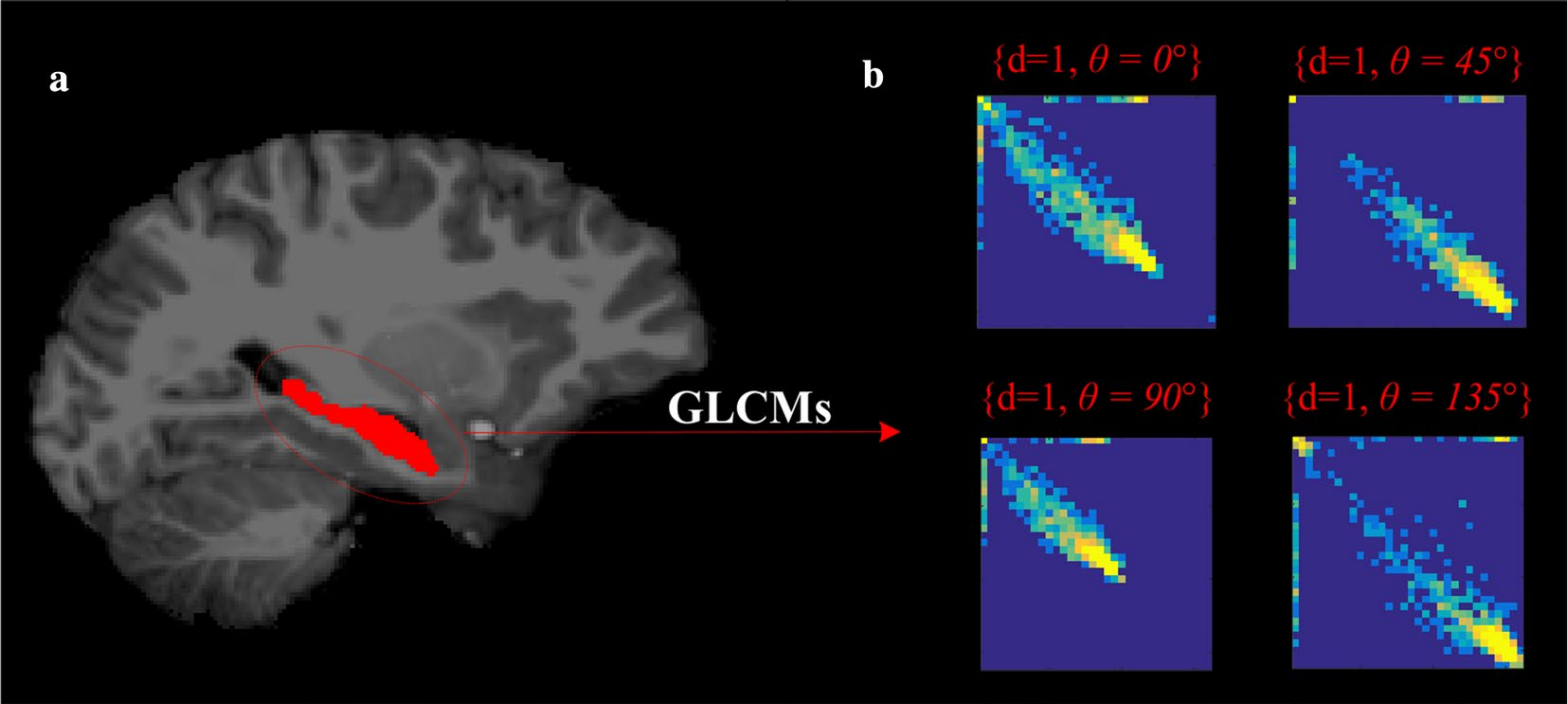
GLCM computation. **a** Labeling of the hippocampus region in *red* (scans reproduced with permission from the International Neuroimaging Data-Sharing Initiative (INDI), under the creative commons license); **b** example of GLCMs corresponding to one offset and four different directions

To obtain GLCM features, we considered the segmented regions corresponding to the hippocampus and amygdala. Intensities within these regions were then equalized to 32 grey levels before computing the GLCM matrices. For every 2D slice containing the region of interest, we computed 4 GLCMs corresponding to offset *d* = 1 and directions *θ* ∈ {0°, 45°, 90°, 135°}. Following this, a set of 11 textures features (or *descriptors*) was obtained for each GLCM by applying to these matrices the following quantifier functions: energy, entropy, correlation, contrast, homogeneity, variance, sum-mean, cluster shade, cluster tendency, maximum probability, and inverse variance. These standard functions are commonly used in radiomic analyses, and capture various properties of tissue heterogeneity [34–39]. The final region representations, composed of 11 features, are obtained by averaging features individually across the four GLCMs and all 2D slices containing the region. Figure 3b shows an example of four GLCMs derived from hippocampal regions (Fig. 3a).

For classification, the resulting features were further processed to have zero mean and unit variance (z-score normalization) [40]:

2 $$x_{\text{norm}} = \frac{{x - \bar{x}}}{\sigma }$$

where *x* is the original feature value, $\bar{x}$ the mean value of this feature, and *σ* its standard deviation.

### Statistical analysis, classification, and validation

An analysis of variance (ANOVA) was first performed on the features extracted from hippocampus and amygdala regions, to compare their distribution in ASD and DC subjects. To account for multiple comparisons (11 texture features = 11 tests), *p* values obtained from this test were corrected according to the Holm–Bonferroni method [41]. We considered texture features with *p* < 0.05 as significant, and selected those for classifying ASD versus DC subjects.

Support vector machine (SVM) models were used for the classification task [42]. Note that other classifiers, such as random forest, have also been tested. However, SVMs provided a superior performance with comparably fewer parameters to tune. For experiments, we considered radial based function (RBF) kernels with a width of *ɣ* = 1, and set the penalty parameter to *C* = 1.

Since the cohort was limited to 28 subjects in group A and 36 subjects in B, a leave-one-out cross-validation strategy was used to obtain a less biased estimate of classification error rates. We evaluated the classification performance using the accuracy, sensitivity and specificity metrics according to the following equations:

3 $${\text{Accuracy}} = \frac{TP + TN }{TP + FP + TN + FN }$$

4 $${\text{Sensitivity}} = \frac{TP }{TP + FN }$$

5 $${\text{Specificity}} = \frac{TN}{TN + FP }$$

where the true positive (*TP*) and the true negative (*TN*) rates are the numbers of correctly classified positive and negative examples. Correspondingly, the false positive (*FP*) and false negative (*FN*) rates are the number of positive and negative examples which are incorrectly classified. Note that we defined DC subjects as positive examples in our experiments.

The performance of our classifier model was also measured using the confusion matrix and area under the ROC curve (AUC). To compute the latter, a 10-fold cross-validation was employed. Specifically, examples corresponding to ASD and DC subjects were randomly divided into 10 folds, each of them used in turn to compute the AUC on remaining samples. The overall performance of the model was then measured as the average AUC obtained over all 10 folds [43].

### Dominant features

Random forest classifiers provide an efficient way to measure feature importance [44]. During the induction phase, discriminative features are selected first when constructing decision trees. In particular, root nodes of decision trees represent the most group-informative features. Following this principle, we used the *TreeBagger* Matlab function to learn a random forest containing 1000 decision trees, for the task of differentiating between ASD and DC subjects. We then measured the importance of features as the frequency at which these features occur in the root node of a decision tree (0–1000 times). Note that feature importance was also measured as the increase in prediction error resulting from permuting features across out-of-bag examples, this strategy giving a similar feature ranking as the one based on root nodes.

### Randomness (permutation) test

Randomness testing is used to quantify the *p* values of individual features and feature combinations [45]. An empirical null distribution is generated from multiple trials, in which subject labels are randomly permutated, thus rendering them information-less regarding the data (i.e. the null hypothesis). Significance *p* values for individual features are then calculated by integrating the tails of the null distribution, based on classification accuracy using true (non-randomized) labels. Addressing the group-wise significance is also important, as a number of features may appear significant due to random chance, particularly in a calculation involving high numbers of features. Techniques such as Bonferroni correction can be overly penalizing, a common alternative is to compute the false discovery rate [46]. In this work, we control the family-wise error rate using the Holm–Bonferroni procedure, which is known to be uniformly more powerful than Bonferroni correction.

## Results

Demographic information (i.e., gender and age) of ASD and DC subjects is provided in Table 1. Except for across-gender age differences in group B, no statistically significant differences in age were found between male and female subjects or between ASD and DC subjects. The age bias in group B could be related to the fact that girls are less likely than boys to meet diagnostic criteria for ASD [47, 48]. The inter-rater reliability of manual segmentation labels is reported in Fig. 2d, showing an average Dice overlap (DSC) above 85% for the hippocampus (left and right), and over 75% for the amygdala (left and right). This confirms the quality of manual labels, in particular for hippocampal regions.

### Feature difference testing

Table 2 gives the average and standard deviation (StDev) of the 11 texture features extracted from the hippocampal and amygdala regions of ASD and DC subjects in the two test groups (i.e., groups A and B). We see significant differences (*p* value <0.05 following Holm–Bonferroni correction) between ASD and DC subjects, in both groups, for all features derived from the hippocampus region. For textures in the amygdala region, 4 texture features (correlation, variance, sum-mean, and maximum probability) showed a significant difference between the ASD and DC subjects of group B. These observations suggest that textures in both the hippocampus and amygdala regions are relevant to ASD, although features derived from the hippocampus may have a greater potential for differentiating between ASD and DC subjects.

**Table 2:** Summary (average ± StDev) of texture features extracted from hippocampal and amygdala regions of ASD and DC patients, with corresponding *p* values

Features	Group A		Group B		*p* value	
	ASD	DC	ASD	DC	Group A	Group B
*Hippocampus*						
Energy	0.990 ± 0.0021	0.992 ± 0.0025	0.599 ± 0.079	0.626 ± 0.049	3.03 × 10 ^−6^	4.21 × 10 ^−4^
Entropy	0.022 ± 0.0044	0.018 ± 0.0053	0.089 ± 0.0019	0.026 ± 0.003	6.96 × 10 ^−6^	8.2 × 10 ^−4^
Correlation	0.488 ± 0.0972	0.584 ± 0.2263	0.735 ± 0.022	0.741 ± 0.015	0.014	0.02
Contrast	0.858 ± 0.1167	0.716 ± 0.1527	1.483 ± 0.492	1.565 ± 0.560	5.24 × 10 ^−8^	7.31 × 10 ^−6^
Homogeneity	0.997 ± 0.0006	0.998 ± 0.0007	0.898 ± 0.024	0.905 ± 0.015	2.31 × 10 ^−5^	8.5 × 10 ^−3^
Variance	2.696 ± 0.5259	2.089 ± 0.6217	9.837 ± 3.311	10.666 ± 4.018	1.31 × 10 ^−6^	7.51 × 10 ^−3^
Sum-mean	1.108 ± 0.0215	1.083 ± 0.0260	3.625 ± 0.463	3.602 ± 0.457	1.76 × 10 ^−6^	8.2 × 10 ^−3^
Cluster shade	476.318 ± 99.8276	369.168 ± 111.025	50.099 ± 21.728	59.828 ± 25.645	2.34 × 10 ^−6^	4.8 × 10 ^−4^
Cluster tendency	23,825.94 ± 5306.88	18,606.34 ± 5536.42	409.330 ± 209.082	507.268 ± 255.621	2.74 × 10 ^−6^	7.12 × 10 ^−5^
Max. probability	0.995 ± 0.001	0.996 ± 0.0012	0.766 ± 0.057	0.786 ± 0.032	3.03 × 10 ^−6^	5.71 × 10 ^−6^
Inverse variance	0.002 ± 0.0004	0.001 ± 0.0004	0.320 ± 0.04	0.407 ± 0.049	9.64 × 10 ^−8^	4.54 × 10 ^−6^
*Amygdala*						
Energy	0.434 ± 0.039	0.422 ± 0.036	0.443 ± 0.043	0.417 ± 0.039	0.317	0.064
Entropy	0.472 ± 0.096	0.473 ± 0.081	0.475 ± 0.112	0.486 ± 0.09	0.985	0.741
Correlation	0.723 ± 0.016	0.728 ± 0.014	0.733 ± 0.021	0.745 ± 0.011	0.389	0.040
Contrast	3.181 ± 0.566	3.366 ± 0.265	2.824 ± 0.459	3.001 ± 0.417	0.235	0.239
Homogeneity	0.855 ± 0.014	0.855 ± 0.011	0.857 ± 0.014	0.859 ± 0.011	0.984	0.602
Variance	19.830 ± 3.414	21.421 ± 1.961	18.433 ± 3.289	20.595 ± 2.854	0.107	0.046
Sum-mean	5.141 ± 0.466	5.357 ± 0.373	4.974 ± 0.503	5.338 ± 0.407	0.141	0.025
Cluster shade	77.687 ± 16.401	80.996 ± 7.505	72.273 ± 13.411	73.692 ± 17.258	0.461	0.783
Cluster tendency	817.399 ± 201.952	884.467 ± 80.220	729.620 ± 166	804.451 ± 181.051	0.220	0.205
Max. probability	0.640 ± 0.035	0.628 ± 0.033	0.649 ± 0.039	0.621 ± 0.038	0.314	0.034
Inverse variance	2.717 ± 0.465	2.878 ± 0.201	2.420 ± 0.380	2.578 ± 0.352	0.207	0.209

### Subject classification

For the classification of typical age range subjects (28 subjects, group A), texture features extracted from the hippocampus yielded a mean accuracy of 67.85%, sensitivity of 64.28% and specificity of 71.42% (Table 3). In the case of non-typical age range subjects (36 subjects, group B), a lower sensitivity of 62.50% was obtained, however the classifier accuracy and specificity increased to 75 and 85%, respectively. By contrast, features derived from the amygdala showed no discriminative power, in both typical and non-typical development groups, with accuracy near 50% (the expected accuracy of a random classifier is 50% for a balanced set of examples).

**Table 3:** Performance metrics (%) of classification between ASD and DC

Subjects	Brain region	Accuracy	Sensitivity	Specificity
Group A (n = 28)	Hippocampus	67.85	64.28	71.42
	Amygdala	52.00	43.75	60.00
Group B (n = 36)	Hippocampus	75.00	62.50	85.00
	Amygdala	50.00	18.75	75.00

*n* number of subjects

Table 4 gives the confusion matrix, summarizing the rates of correct and incorrect SVM classification for ASD and DC subjects. Using hippocampus texture features, a correct classification was achieved for 10/14 ASD and 9/14 DC typical age range subjects, compared to 17/20 ASD and 10/16 DC non-typical age range subjects. This difference in accuracy may be due, in part, to the class imbalance in group B (20 ASD vs 16 DC). Compared to hippocampus, amygdala texture features lead to lower classification rates for ASD and DC subjects, in both groups A and B.

**Table 4:** Summary of confusion matrix

Subjects	Hippocampus				Amygdala			
	Group A (n = 28)		Group B (n = 36)		Group A (n = 28)		Group B (n = 36)	
	ASD (14)	DC (14)	ASD (20)	DC (16)	ASD (14)	DC (14)	ASD (20)	DC (16)
ASD	10	4	17	3	12	8	15	5
DC	5	9	6	10	9	7	13	3

*n* number of subjects

**Fig. 4:**
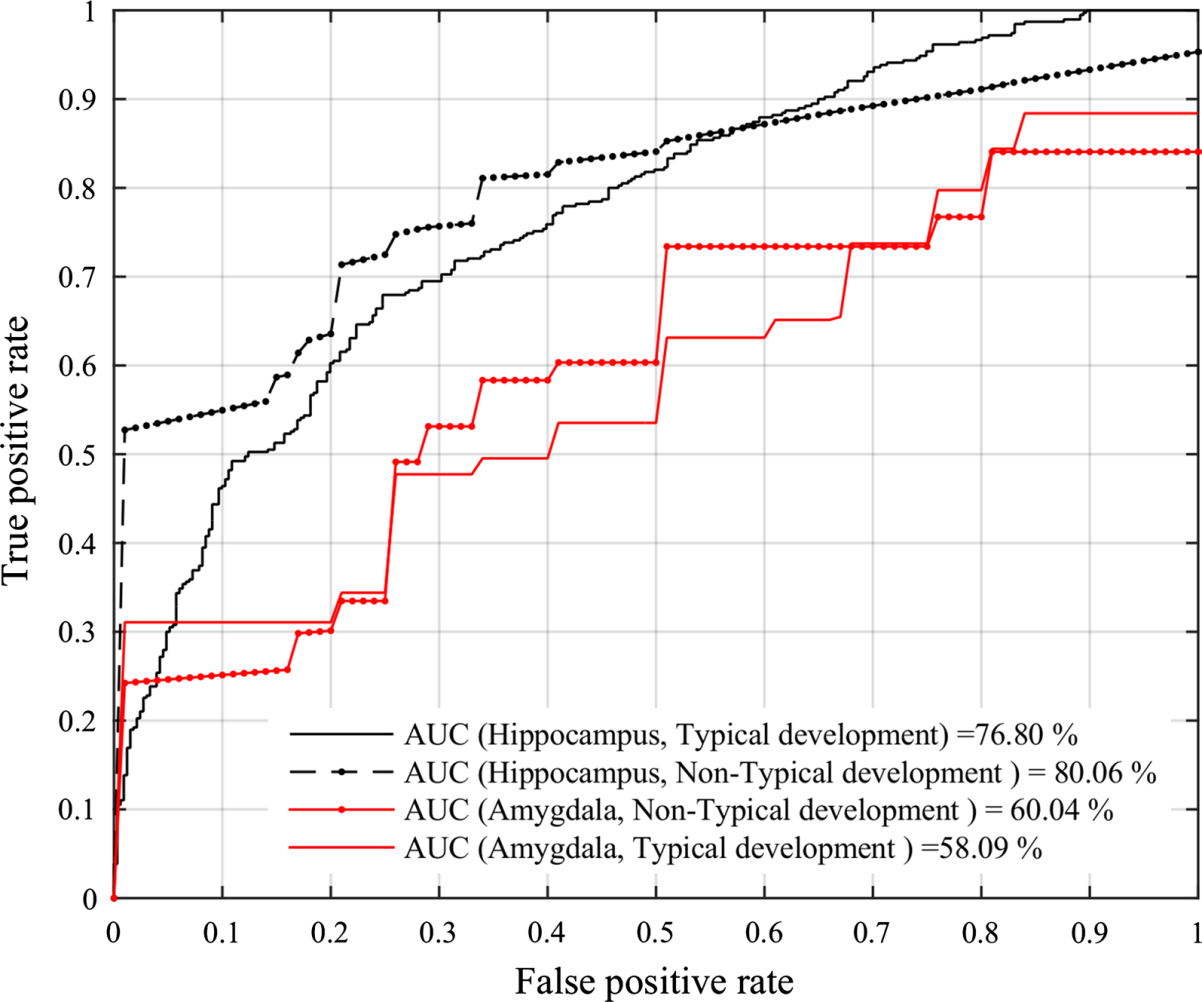
ASD versus DC classification performance. Mean receiver operating characteristic (ROC) curve and AUC obtained by the SVM using the texture features derived from hippocampus (*black curves*) and amygdala (*red curves*) regions in typical (group A) and non-typical (group B) age range subjects

Figure 4 shows the mean ROC curves and AUC values obtained by the SVM model for classifying typical and non-typical age range subjects, using texture features from the hippocampus (black curves) and amygdala (red curves) regions. Once again, it can be seen that features derived from the hippocampus lead to a better performance than those extracted from the amygdala, with a mean AUC of 76.80 and 80.06% compared to 58.09 and 60.04%, respectively for typical and non-typical age range subjects. Results of the randomness test are reported in Table 5. As expected, the null distribution of classification accuracy peaks around 50% for hippocampus and amygdala features, in both subject groups. This confirms that the results obtained with the proposed texture features are not due to chance.

**Table 5:** Summary of permutation test

Brain regions	Subjects	Average ± StDev	Median
Hippocampus	Group A	48.10 ± 12.78	50.00
	Group B	52.00 ± 8.50	51.11
Amygdala	Group A	49.90 ± 9.62	51.20
	Group B	51.34 ± 6.32	50.36

*StDev* standard deviation

### Feature importance

**Fig. 5:**
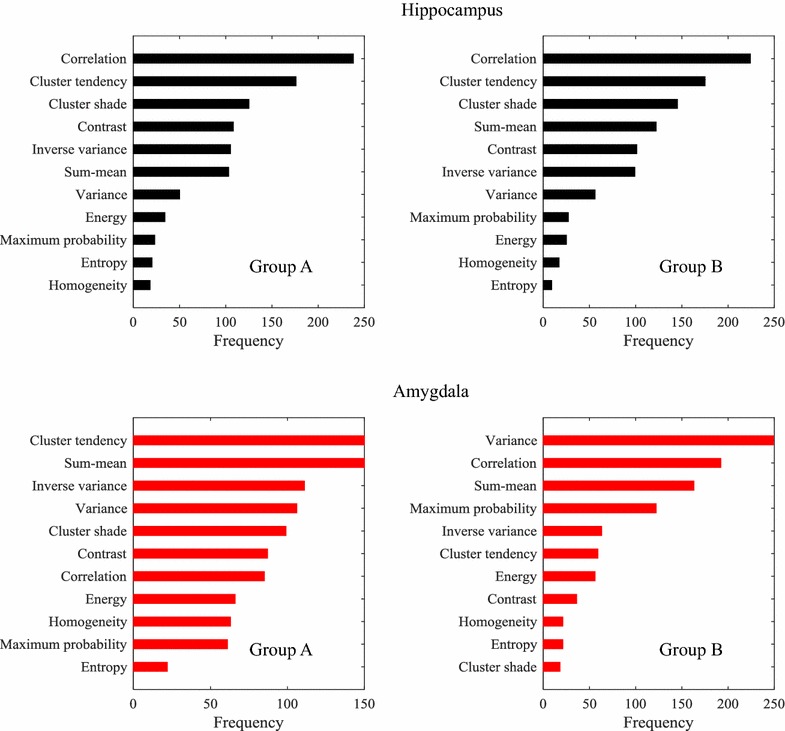
Dominant feature identification. (*First row*) Hippocampus-derived features; (*second row*) amygdala-derived features. Each *bar* represents the occurrence number of a feature in decision-tree root nodes (from 0 to 1000). Group A (*left*) and group B (*right*) contains typical and non-typical age range subjects, respectively

Figure 5 gives the relative importance of features derived from the hippocampus and amygdala regions, in subject groups A and B, as measured by the number random forest trees having these features as root node. For the hippocampus, we find that the correlation, cluster tendency, cluster shade, contrast, inverse variance, and sum-mean features are the most dominant, with 100 or more root node occurrences in both groups A and B. All other features have less than 100 occurrences. Moreover, it can be seen that the feature ranking is consistent across subject groups, with correlation being the most informative feature in the two groups. In contrast, the importance of features extracted from the amygdala varies more significantly from group A to group B. This supports the results of our previous analyses, which found texture in the amygdala to be less relevant than that of the hippocampus for identifying ASD subjects.

## Discussion

Radiomic features, and particulary those encoding texture, enable the quantification of voxel (or pixels) inter-relationships, describing characteristics of underlying tissues that may be invisible to the human visual system [49]. For example, texture features can help segment lesions in glioblastoma multiforme [50]. Likewise, abnormal textures in the corpus callosum and thalamus were found to be associated with Alzheimer’s disease [51]. Abnormal texture patterns in the pons, midbrain, dentate nucleus, globus pallidus, and corona radiata can also be observed in subjects with Parkinson’s disease [52]. However, the link between texture in neuroanatomical regions and ASD has so far been unclear.

In this study, GLCM-based texture features derived from the hippocampus (11 features) and amygdala (11 features) regions were used for differentiating between ASD and DC subjects. Note that the gender differences in the untypical age group (i.e. group B) could be related to pathophysiological reason and almost the ASD is approximately 3–4 times more prevalent in boys than girls [53]. The first analysis, using ANOVA, found 11 hippocampi (groups A and B) and 4 amygdalae (group B) features that were significantly different in ASD subjects compared to DC subjects (Table 2). Various studies in the literature have reported abnormal brain development curves for ASD subjects, which may lead to volumetric differences in structures like the hippocampus [16, 54]. It is possible that this abnormal development affects the underlying substrate, thereby leading to the observed differences in texture.

Our classification analysis based on SVM showed a higher performance (accuracy and AUC) of texture features from the hippocampus than those derived from the amygdala, with a mean accuracy of 67.85% and mean AUC of 76.80% in the 28 typical age range subjects, where ASD and DC subjects were matched based on age (Fig. 4; Table 3). This suggests that hippocampus texture features could be used effectively as biomarkers for detecting ASD. In particular, our feature importance analysis based on random forests indicated hippocampus GLCM correlation to be the most discriminative feature for differentiating between ASD and DC subjects (Fig. 5). This feature measures the linear dependency of grey levels between neighboring pixels, and is related to region heterogeneity (e.g., correlation is 0 for a completely uniform region).

Our findings on the non-difference of amygdala texture features between ASD and DC subjects (group A) are consistent with previous studies showing no significance difference in amygdala volume between ASD and DC subjects [55]. Although other studies have reported an enlarged amygdala in ASD subjects [16, 56, 57], the differences observed for non-typical age range subjects (group B) could be due to the age bias from using non-matched ASD and DC subjects.

Our proposed approach differs from traditional techniques, which mostly rely on morphological and volumetric characteristics [16–18]. Research suggests that the white matter in young children with ASD may be abnormally homogeneous, and this may reflect poor organization or differentiation of pathways in the temporal lobe [58]. Another study using multimodality neuroimaging (e.g. structural MRI, diffusion tensor imaging and proton magnetic resonance spectroscopy) found that ASD subjects had alterations in cortical thickness, white matter connectivity, as well as neurochemical concentration, demonstrating the potential of multimodal imaging as a more informative method to identify ASD [59].

Moreover, a recent study demonstrated that the differences between ASD and DC may depend on acquisition site. This study suggested applying a significance-weighted principal component analysis (PCA) technique to reduce the undesired intensity variance, thereby increasing the statistical power in detecting the differences between ASD and DC groups [60]. Using this technique, Broca’s area and temporal-parietal junction were found to be significantly different. However, the classifier accuracy between ASD and DC was not sufficient to classify diagnostic groups. Nevertheless, this study motivated our decision of using data from a single site, instead of all available sites, to avoid introducing cross-site intensity variance in our analysis of texture. Other studies have argued that MRI techniques are too spatiotemporally limited to appreciate the synaptic or neuronal-level abnormalities that may be at the of disorders like ASD [61]. Our work suggests that MRI texture, which stem from tissue heterogeneity, could capture these abnormalities at a higher scale and, thus, be used for understanding ASD. A broader investigation involving more subjects would however be required to clarify the nature of texture differences and their impact on ASD.

Our study has several limitations worth of mention. The number of subjects (i.e., 64) is relatively low, especially in the case of ASD where most subjects are at a developing age. In the proposed methodology, age bias was addressed by matching ASD and DC subjects. However, the primary goal of this study was to assess the feasibility of using texture features derived from neuroanatomical region for discriminating between the ASD and DC subjects. Furthermore, this study was limited to T1-WI MRI data only. Additional information could be gained by considering the texture features computed from different, complementary MRI sequences, such as T1-WI pre- and post-contrast, T2-WI, or FLAIR. Moreover, employing more advanced methods for segmenting brain regions and classification, for instance based on deep learning, could potentially increase the performance of our approach.

## Conclusions

This paper presented a radiomic approach using GLCM texture features derived from hippocampal and amygdala regions to characterize differences between ASD and development control subjects. Our preliminary results show the potential of these features as a biomarker to aid clinicians in the diagnostic of ASD. Texture features derived from the hippocampus, and particularly GLCM correlation, were found to have significant discriminative power for differentiating between ASD and control subjects. Future work can include a validation of the proposed approach on a larger subject cohort, and using additional imaging modalities.

## Abbreviations

ASD: autism spectrum disorder
DC: development control (normal patient)
GLCM: grey level co-occurrence matrix
